# Effects of geography on risk for suicidal ideation and suicide attempts among commercially insured children and youth in the US

**DOI:** 10.1192/j.eurpsy.2023.281

**Published:** 2023-07-19

**Authors:** J. Pathak

**Affiliations:** Population Health Sciences, Cornell University, New York, United States

## Abstract

**Introduction:**

To study the effects of geography on risk for suicidal ideation and suicide attempts among commercially insured children and youth in the US

**Effects of geography on risk for suicidal ideation and suicide attempts among commercially insured children and youth in the US**

**Objectives:**

Few studies have examined the impact of geography on risk factors for suicidal ideation (SI) and suicide attempts (SA). This study used a national representative sample to study how geography may influence the relationships of risk factors for SI and SA in commercially insured children and youth.

**Methods:**

The sample was a nationwide retrospective cohort study of 124,424 patients <25 years using commercial claims from four major insurance companies (Aetna, Humana, Kaiser Permanente, and UnitedHealthcare) in the US. The index visit was a mental health or substance use (MH/SUD) outpatient encounter between January 2014 and June 2015. SI and SA were defined by having an ICD-9 diagnosis code within one year after the index visit. Risk factors in the models were demographic and clinical risk factors, including prior psychiatric diagnoses, prescriptions, and healthcare services utilization. Patients’ geographic regions were assigned to one of the nine divisions defined by the US Census Bureau. We used survival analysis to evaluate the effects of geography on risk factors for SI and SA.

**Results:**

At each follow-up time period (post 7-, 30-, 90-, 180-, and 365-day), rates of SI and SA varied by geographic division (p<0.001). The Mountain Division consistently had the highest rates for both SI and SA (5.44%-10.26% for SI; 0.70%-2.82% for SA). Having MH emergency department (ED) visits in the past year increased the hazard ratio of SI by 28%-65% for children and youth residing in the New England, Mid-Atlantic, East North Central, West North Central, and East South Central Divisions. The main effects of geographic divisions were significant for SA (p<0.001). Risk of SA was lower in New England, Mid-Atlantic, South Atlantic, and Pacific (HRs=0.57, 0.51, 0.67, and 0.79, respectively) and higher in the Mountain Division (HR=1.46).

**Conclusions:**

Children and youth residing in the Mountain Division had the highest prevalence of SI and SA and the highest risk of SI after having MH ED visits. Studies of indicators of access to MH ED care and other social determinants of health may clarify the reasons for SI and SA geographic differences.

**Disclosure of Interest:**

None Declared

